# Comparison of Standard Setting Procedures to Establish Defensible Passing Standards for Clinical Skills Assessment: Angoff, Borderline Group, Contrasting Groups and Patient Safety Methods

**DOI:** 10.1111/tct.70198

**Published:** 2025-09-03

**Authors:** Rachel S. Tappan, Megan L. Freeland, Elizabeth E. Holland, Yoon Soo Park, Heidi R. Roth

**Affiliations:** ^1^ Department of Physical Therapy and Human Movement Sciences, Feinberg School of Medicine Northwestern University Chicago Illinois USA; ^2^ Department of Medical Education, College of Medicine University of Illinois Chicago Illinois USA

**Keywords:** assessment, clinical competence, competency assessment, health professions education, physical therapy education, standard setting

## Abstract

**Introduction:**

Standard setting methods for clinical skills assessments help establish cut scores that accurately reflect clinical performance expectations. However, these methods lead to varied cut scores, and guidance for method selection is limited. This study compares the application of four methods.

**Methods:**

The Angoff, Patient Safety, Borderline Group and Contrasting Groups methods were applied to an assessment of physical therapist student clinical skills. The resulting cut scores were applied to a de‐identified historical dataset (*n* = 92). Post hoc logistic regression analysis evaluated the underlying constructs for global ratings of student competence.

**Results:**

The cut scores ranged from 65.9% to 86.1%, with the Angoff method resulting in the lowest cut score and the Borderline Group method resulting in the highest cut score. Applying the cut scores to the historical dataset resulted in pass rates ranging from 60.9% to 98.9%. Logistic regression modelling revealed that increasing Safety Score was associated with an increased likelihood of receiving a ‘pass’ global rating with an odds ratio of 18.97 (95% CI, 2.30–156.63). Total Score did not have a statistically significant association.

**Conclusion:**

The Patient Safety method required a higher performance level for items related to safety, aligned with expert judges' conceptualisation of competence. Therefore, the Patient Safety method demonstrated the best match for this assessment's goals. These findings can inform method selection for clinical skills where patient safety is a key consideration. Additional recommendations are to include pass rate feasibility and conceptual alignment with the target construct when selecting standard setting methods.

## 1 Introduction

To fully serve health professions trainees, patients and society, educational assessment decisions about minimum passing standards must accurately reflect learners' readiness for patient care. Multiple standard setting methods exist in the health professions education literature to inform such decisions. Although different standard setting methods may yield large variations in cut score [1], guidance for selecting among these methods is limited. In this study, we apply four standard setting methods to a clinical skills assessment and generate recommendations for standard setting method selection based on the results.

The *Standards for Educational and Psychological Testing* state that ‘validity refers to the degree to which evidence and theory support the interpretation of test scores for proposed uses of tests’ [2, p. 11]. This definition suggests that assessment interpretation depends on the proposed use and context of the assessment. Therefore, setting an appropriate assessment cut score (i.e., the minimum passing standard) may vary depending on factors, including the skill being tested, level of training, expected level of competence and clinical setting. For clinical skills assessment, making sound pass/fail decisions is essential for determining whether a learner is ready to perform clinical skills with actual patients.

*For clinical skills assessment, making sound pass/fail decisions is essential for determining whether a learner is ready to perform clinical skills with actual patients.*

Standard setting methods for determining assessment cut scores are a meaningful source of consequential validity evidence [3, 4, 5]. Different methods may result in widely varied passing standards [6, 7]. Therefore, method selection is of critical importance. The health professions education literature offers many methods for standard setting (see Table 1) but less guidance for method selection [6, 8, 9, 10, 11, 12, 13, 14, 15, 16]. Existing guidance suggests that the selected method should be aligned with the goals of the assessment, feasible to implement and explain to users and evidence‐based [12]. However, the description of how to accomplish these recommendations is limited.

**TABLE 1:** List of standard setting approaches commonly used in performance‐based assessments with a description of each approach.

Approach	Examinee‐ or test‐centred	Norm‐ or criterion‐referenced	Description
Angoff [8, 9]	Test	Criterion	Judges rate the percentage of minimally competent learners that would perform each item correctly Cut score is the mean of all ratings across all judges and items
Borderline Group [8, 9]	Examinee	Criterion	Examinees are scored with a multiple‐item assessment tool and with a global rating scale indicating whether the learner passed, marginally passed/failed or failed the assessment Cut score for the checklist is then determined by the scores of the examinees who received a global rating of marginally pass/fail
Contrasting Groups [8, 9]	Examinee	Criterion	Examinees are scored with a multiple‐item assessment and with a global rating scale indicating whether the examinee passed or failed the assessment Cut score is the score that best discriminates between examinees with global ‘pass’ rating and those with global ‘fail’ rating
Ebel [8, 9]	Test	Criterion	Items are organised based on relevance (according to ratings from a panel of judges) and difficulty (according to assessment results from previous learners) Judges estimate the percentage of minimally competent learners who will successfully complete the items Cut score is based on a weighted mean of these ratings that takes item relevance into account
Hofstee [6, 8, 9, 10]	Test	Norm and criterion	Judges provide minimum and maximum acceptable passing scores and failure rates These criteria are then applied to learner performance data to determine the cut score
Mastery Angoff [6, 10]	Test	Criterion	Same as Angoff method, except the criterion is ‘well prepared’ rather than ‘minimally competent’ learners
Patient Safety [6, 8, 11]	Test	Criterion	Judges identify items with essential aspects of performance (safety, outcome and comfort) and set a separate minimum passing standard for essential and non‐essential items

The purpose of this study is to compare the application of multiple standard setting methods to a clinical skills assessment to determine which method best aligns with the proposed assessment use. Specifically, the study aims to (1) compare the application of the Angoff [8, 9], Patient Safety [8, 11], Borderline Group [8, 9] and Contrasting Groups [8, 9] methods to an assessment of student physical therapists' (SPTs') clinical skill performance; (2) evaluate the consequences of these methods on pass rates for this assessment using historical data from a Doctor of Physical Therapy (DPT) programme; and (3) investigate the underlying factors that influence expert judges' evaluation of student competence. Although this study uses an assessment from DPT education, the considerations for standard setting method selection in clinical skills assessments may be similar across health professions. Therefore, the results of this study will provide evidence to inform standard setting method selection in clinical skills assessments across health professions.

## 2 Methods

In this cross‐sectional study, the number of student cases was determined by the student cohort size. The number of standard setting judges was based on guidelines recommending a range of four to 20 [12].

### 2.1 Assessment Interpretation and Use

The bed mobility clinical skills assessment is embedded within a foundational skills course in the penultimate trimester preceding the first full‐time clinical experience (ce1) during the first year of a 3‐year DPT programme at Northwestern University. During ce1 and subsequent coursework, SPTs must be able to safely and effectively evaluate and treat patients performing functional mobility tasks (e.g., moving in bed, transferring from one surface to another and locomotion) [17]. The assessment's purpose is to determine whether the SPTs are ready to effectively and safely examine, assist and train patients in bed mobility tasks including rolling, transitioning between sitting and supine and positioning in bed during ce1. The assessment requires each SPT to assist a standardised patient with each task and uses a previously published 48‐item checklist with a dichotomous (‘Yes’/‘No’) rating scale (Appendix S1a) [18]. The Total Score is determined by the percentage of items with a ‘Yes’ rating.

### 2.2 Standard Setting

Criterion‐referenced standards are most appropriate when the goal is for everyone to eventually achieve competent skill performance [19]. Therefore, we selected criterion‐referenced methods for this study: the Angoff, Patient Safety, Borderline Group and Contrasting Groups methods [8, 9, 11, 12, 13, 14]. Other commonly used criterion‐referenced methods that also incorporate absolute and relative standard setting, such as the Hofstee method, were not included because relative standard setting procedures are not consistent with the assessment and curricular goal for all learners to eventually achieve competent performance.

**FIGURE 1:**
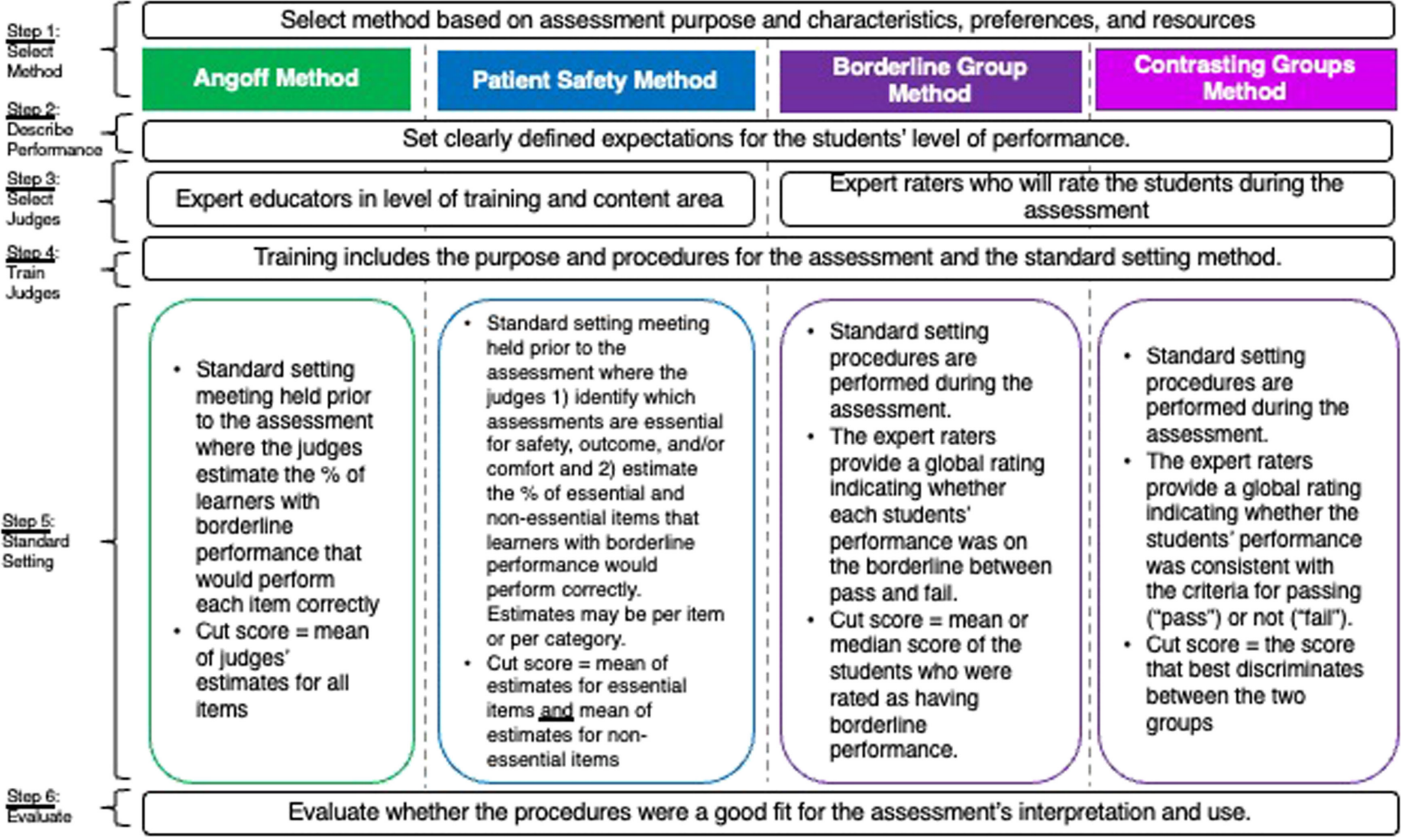
Overview of the procedures for the Angoff, Patient Safety, Borderline Group and Contrasting Groups standard setting methods.

See Figure 1 for an overview of the steps for the methods included in this study and Appendix S2 for a detailed account of the steps applied. Based on the assessment's proposed interpretation and use, expectations for passing this assessment were that SPT performance would be consistent with a minimally competent level for entrance into ce1.

### 2.3 Angoff Method

We selected the Angoff method because of its widespread use in health professions education and ease of administration [9].

The judges were physical therapists with experience teaching first‐year SPTs from Northwestern University DPT programme in the classroom or the clinic. Judge selection aimed for diverse representation of teaching roles and experience in clinical settings where patients frequently require assistance with bed mobility tasks.

The standard setting procedures occurred during a two‐hour online meeting using Zoom video conference software (Zoom Video Communications Inc., San Jose, CA). The judges answered the following question for each checklist item, ‘What is the likelihood (0‐100%) that a minimally competent (i.e., borderline) learner entering CE1 would perform each item correctly?’ The mean of the judges' final ratings was the cut score applied to the Total Score.

### 2.4 Patient Safety Method

We selected the Patient Safety method because it was developed for use with clinical skills assessments, where some items (the ‘essential’ items) are more important than others and therefore held to a higher standard than the ‘non‐essential’ items [11]. We felt that the incorporation of considerations for patient safety aligned well with clinical and accreditation priorities of patient safety [20, 21, 22, 23, 24].

We determined a priori that the essential items would be those that were essential for *patient safety*. The same judges participated in the Patient Safety method and the Angoff method procedures during the 2‐h online meeting. The judges rated which checklist items were essential according to the following criterion: ‘Critical items where patient safety would be at risk if the student performed the item inconsistently, ineffectively, or not at all’ with 100% agreement among judges required for an item to be named an ‘essential item’. All other items were non‐essential.

Next, judges determined separate cut scores for essential and non‐essential items by answering the following questions:

1. What percentage of essential/critical safety items would a minimally competent learner entering CE1 perform correctly?
2. What percentage of non‐essential items (i.e., items not critical for patient safety) would a minimally competent learner entering CE1 perform correctly?

Two conjunctive cut scores (i.e., with separate cut‐off scores for the essential items and non‐essential items) were calculated via two methods: (1) *category‐based method*, calculating the mean of the judges' answers to the two questions listed above, and (2) *item‐based method*, calculating the mean of the judges' item‐by‐item estimates from the Angoff standard setting process.

### 2.5 Borderline Group and Contrasting Groups Methods

We selected the Borderline Group and Contrasting Groups methods because of their incorporation of learners' actual performance in the standard setting process and widespread use in health professions education [8, 9].

The judges were the faculty assessors who rated SPT performance during the assessment using the assessment checklist. Immediately after each SPT completed the assessment as part of the standard curriculum, the faculty assessor provided global ratings of the SPT's overall performance by answering the following questions: (1) Would you rate this student's performance as ‘pass’ or ‘fail’? (2) Was this student's performance on the borderline between pass and fail, that is, ‘marginal pass’ or ‘marginal fail’? For the Borderline Group method, the cut score was the mean Total Score of the SPTs who received a rating of ‘borderline’ performance in the first question. For the Contrasting Groups method, the score distributions for the students with a rating of ‘pass’ and those with a rating of ‘fail’ in the second question were graphed. The cut score was set at the intersection of the two distributions.

### 2.6 Evaluation of Cut Scores

To understand which of the four standard setting methods was most suitable for the intended interpretation and use of this assessment, we evaluated the feasibility of the cut scores as well as the factors contributing to the judges' ratings. We investigated cut score feasibility by calculating the pass/fail rate that would have resulted from each cut score using de‐identified assessment data from a previous single cohort of 92 SPTs. Assessment failures carry potential consequences for learner progression in the programme. Therefore, evaluating the pass/fail rate associated with the cut score based on a previous cohort provides feasibility evidence for the cut score by providing an estimate of future impact on learners, assuming future performance remains stable.

Variability in cut score results among the four methods led us to question whether the standard setting procedures were reflective of the same or different underlying constructs. A notable aspect of the Borderline Group and Contrasting Groups methods is that they connect the judges' global ratings of learner performance to learners' test scores. However, **what if the faculty judges' conceptualisation of competence for learners' first‐time clinical experience (the target of the global rating) was not primarily based on each learner's overall performance?** We hypothesised that at the SPTs' early stage of training in our study, the judges' perceptions of their *safety* during the patient care task (e.g., avoiding fall risk and not pulling on lines connected to the patient) may be more influential than their *total performance*, which includes additional elements such as effectiveness, communication and documentation. This hypothesis was supported by statements that judges made during the standard setting meeting, such as a comment that more than one safety error would indicate a less than minimally competent performance at this stage in training. Therefore, we conducted a post hoc analysis to explore further.

*Variability in cut score results among the four methods led us to question whether the standard setting procedures were reflective of the same or different underlying constructs.*

## 3 Data Analysis

During the Angoff and Patient Safety procedures, descriptive statistics (mean, SD and range) of judges' ratings were calculated. During the Patient Safety procedures, the frequencies of ‘Yes’ and ‘No’ ratings were calculated to determine essential items. The cut scores for the Angoff and Patient Safety methods were calculated as the mean of judges' ratings in real time during the standard setting meeting. Each cut score was applied to de‐identified historical data, and descriptive statistics (mean scores, frequency of pass/fail decisions) were calculated.

Post hoc analysis investigated the relationship between the SPTs' global ratings of ‘pass’/‘fail’ from the Borderline Group procedures and the SPTs' checklist scores by graphing the global ratings against the total checklist scores (Total Score) and the scores on the essential items identified during the Patient Safety procedures (Safety Score). We calculated a binary logistic regression model to examine the odds of predicting ‘pass’ global ratings using the Total and Safety Scores and report model significance, model fit and odds ratios with 95% confidence intervals.

During the standard setting meeting, data compilation and descriptive analyses were conducted with Google Sheets software (Google, Mountain View, California). We used IBM SPSS Statistics for Mac, Version 29.0.2.0 (IBM Corp., Armonk, New York) to conduct inferential statistics. The Northwestern University Institutional Review Board approved this study (STU00220525), and all participants (the standard setting judges) provided online written consent.

## 4 Results

### 4.1 Standard Setting Outcomes

See Table 2 for characteristics of the standard setting judges. One participant was withdrawn from the study due to an incomplete dataset.

**TABLE 2:** Characteristics of the standard setting judges.

Judge #	Standard setting panel	Education role with first‐year DPT students	# of years as a PT	# of years in any education role	Current clinical practice setting
1	Angoff/Patient Safety	Associated faculty	7	2	Inpatient rehabilitation
2	Angoff/Patient Safety	Associated faculty	11	1	Inpatient rehabilitation
3	Angoff/Patient Safety	Core faculty	46	37	Not applicable
4	Angoff/Patient Safety	Core faculty	20	11	Outpatient
5	Angoff/Patient Safety	Clinical Instructor	3	1	Day Rehabilitation
6	Angoff/Patient Safety	Core faculty	29	15	Not applicable
7	Angoff/Patient Safety	Associated faculty	24	15	Acute Care
8	Angoff/Patient Safety	Associated faculty	7	3	Inpatient rehabilitation
9	Angoff/Patient Safety	Associated faculty and Clinical Instructor	9	3	Inpatient rehabilitation
10	Borderline Group	Associated faculty	8	1	Inpatient rehabilitation
11	Borderline Group	Core faculty	26	17	Research
12	Borderline Group	Associated faculty	25	9	Outpatient
13	Borderline Group	Associated faculty	23	10	Acute care
14	Borderline Group	Associated faculty	14	9	Home health
15	Borderline Group	Associated faculty	13	5	Acute care
16	Borderline Group	Associated faculty	13	9	Acute care
17	Borderline Group	Core faculty	21	10	Outpatient
18	Borderline Group	Associated faculty	18	16	Research
19	Borderline Group	Core faculty	41	23	Acute care

*Note:* Core faculty are employees of the DPT programme whose primary role is to teach in the DPT programme. Associated faculty are clinicians employed by outside clinics who assist with teaching as lab assistants, assessors and guest lecturers. Clinical instructors are clinicians who supervise students during full‐time clinical experiences.

Appendix S1a outlines the mean rating for each item during the Angoff method and the frequency of ‘Yes’ ratings for each checklist item during the Patient Safety method. Appendix S1b lists the 14 checklist items identified as essential during the Patient Safety method process. In the Borderline Group method, 12 out of 85 SPTs were rated as having a borderline performance. In the Contrasting Groups method, 73 SPTs received a rating of ‘pass’, and 12 SPTs received a rating of ‘fail’. Table 3 lists the calculated cut scores from each standard setting method.

**TABLE 3:** Cut scores from the current study and hypothetical pass rates based on retrospectively applying each standard to a historical dataset of de‐identified assessment data.

Method	Minimum passing standard (cut score in %)	Historical pass rate
Angoff	Total score: 67.8%	98.9% (91/92 students)
Patient Safety—category‐based	Essential items: 85.0% Non‐essential items: 66.1%	96.7% (89/92 students)
Patient Safety—item‐based	Essential items: 72.5% Non‐essential items: 65.9%	97.8% (90/92 students)
Borderline Group	Total score: 86.1%	60.9% (56/92 students)
Contrasting Groups	Total Score: 83.3%	71.7% (66/92 students)

### 4.2 Cut Score Evaluation

#### 4.2.1 Feasibility

In the historical dataset, the mean assessment Total Score was 88.1% (SD 6.9%), the mean Safety Score was 95.9% (SD 6.5%), and the mean score on the non‐essential items was 84.9% (SD 8.9%). See Table 3 for pass rates when each method's cut score was applied to the historical dataset.

#### 4.2.2 Post Hoc Analysis of Underlying Constructs

**FIGURE 2:**
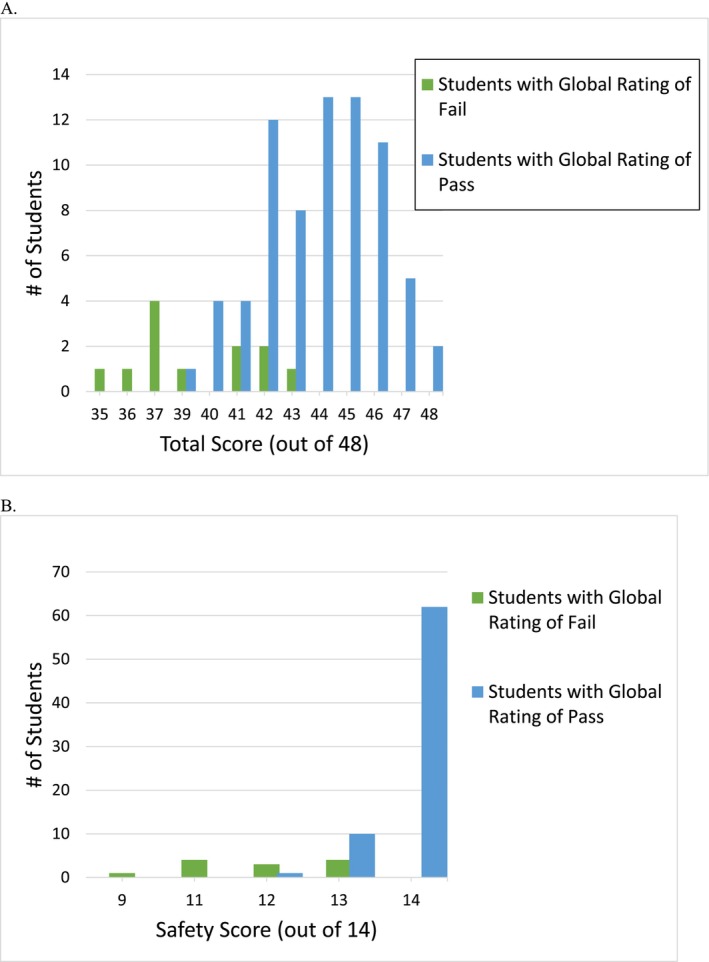
(A) Number of student physical therapists (SPTs) rated with global rating of ‘pass’ and ‘fail’ by Total Score. (B) Number of SPTs rated with global rating of ‘pass’ and ‘fail’ by Safety Score.

Graphing the SPTs' Total Scores and Safety Scores against their ‘pass’ and ‘fail’ global ratings revealed less overlap in Safety Scores between the SPTs rated as ‘pass’ and those rated as ‘fail’ compared to the Total Scores (see Figure 2).

The logistic regression model to predict the global rating outcome was statistically significant [*X* ^2^(2) = 48.988, *p* < 0.001], with a pseudo *R* ^2^ of 0.786 (Nagelkerke *R* ^2^), and correct classification of 92.9% of cases. Model estimates suggest that each one‐point increase in Safety Score was associated with 18.97 times higher (95% CI, 2.30–156.63) likelihood of receiving a ‘pass’ global rating. Total Score did not have a statistically significant association, with an odds ratio of 1.83 (95% CI, 0.85–3.95).

## 5 Discussion

### 5.1 Main Findings

In this study, we applied the Angoff, Patient Safety, Borderline Group and Contrasting Groups standard setting methods to a clinical skills assessment of SPTs. We then evaluated the feasibility of the cut scores as well as the factors contributing to the judges' ratings to understand which method was most suitable for the intended interpretation and use of this assessment. Several studies have previously compared the application of different standard setting methods to an assessment in health professions education [6, 7, 15, 25]. This study extends that previous work by using post hoc analysis to investigate the factors contributing to the judges' conceptualisation of competence, which may not be readily apparent. Previous studies that directly compare standard setting methods have shown variation in cut scores between methods [6, 15], though not as high as the range of 20.2% found in this study.

Each method included in this study has advantages and disadvantages. The Angoff method was simple and efficient to implement and resulted in a cut score that would have led to a high—and therefore feasible—pass rate in a previous cohort. However, a disadvantage is that the single cut score allows learners to compensate for low performance in some areas with high performance in other areas [11]. As a result, learners may pass the assessment, despite not correctly performing items that are essential for patient safety or outcomes.

The Borderline Group and Contrasting Groups methods resulted in the most stringent cut scores and therefore the lowest pass rates. As a result, these cut scores may have the highest sensitivity for identifying learners who are not ready to advance in their training. However, the low pass rates (60.9% and 71.7%, respectively) seen in the historical dataset indicate that the more stringent cut scores could cause substantial learner and faculty burden and therefore may not be feasible. These methods also allow learners to compensate for low performance in some areas with high performance in other areas [11]. The implementation of these methods was resource‐efficient because they did not require a separate standard setting meeting. The Borderline Group method requires a relatively large number of students receiving a ‘borderline’ rating, which may be a limitation of this method in some settings.

An advantage of the Patient Safety method is that the more stringent cut score for essential items ensures that learners do not pass the assessment if they have unsafe performance that could result in adverse patient outcomes [11]. In this study, only the Patient Safety method included consideration for weighting items related to safety more heavily, which was well aligned with the post hoc analysis suggesting that the judges' evaluation of readiness for ce1 was heavily influenced by the SPT's ability to interact with a patient *safely*. The Patient Safety method also resulted in a high pass rate, increasing its feasibility. Disadvantages are that the Patient Safety method involves a more detailed, multi‐step process and a more complex cut score, which may require additional preparation.

The Patient Safety method was the best match for the intended use of this assessment because it (1) resulted in a cut score that was feasible based on previous learner performance and (2) generated a standard for passing that emphasised safe performance, which aligned with the judges' conceptualisation of competent performance at this stage of training. The Patient Safety method is a relatively new standard setting method that has not been reported as often as the other methods in this study. As clinical skills often have some components that are more critical than others for successful patient care outcomes, we believe that the Patient Safety method merits wider implementation.

*As clinical skills often have some components that are more critical than others for successful patient care outcomes, we believe that the Patient Safety method merits wider implementation.*

### 5.2 Implications for Health Professions Education

Standard setting methods support educators' assessments of learner readiness for patient care by providing validity evidence for the decisions made as a consequence of clinical skills assessment [4, 5, 26]. Although this study centred around an assessment of physical therapy clinical skills, the results can inform standard setting method selection for similar clinical skills assessments across the health professions. Based on these study findings, we recommend that educators consider two important sets of questions to guide standard setting method selection:

1. **What constructs underlie competence for the proposed interpretation and use of the assessment? Which standard setting method best aligns with the underlying construct(s)?**
  Educators can identify these underlying constructs with theoretical or empirical evidence. In this study, we hypothesised that safety was an important component of competence for this assessment, which was supported by standard setting judges' comments. We also investigated the judges' conceptualisation of competence empirically through post hoc statistical analysis. Other methods to investigate the constructs that influence the evaluation of competence for the target skills include interviews, focus groups and surveys with faculty, clinicians, patients and/or learners.
  These underlying constructs may vary based on context and timing. For instance, for the assessment in this study, safety was the major determining factor for competence early in training. Later in training, it is possible that the importance of learner effectiveness may be equally or more important. The expected level of competence may also be relevant for standard setting method selection. For instance, the Patient Safety method will be an appropriate choice when safety is a factor in deciding whether a learner is *minimally competent*. Additional factors may become important for determining whether a learner is *well prepared*. In that case, a variation of the Patient Safety method where additional aspects of the skill (e.g., effectiveness or patient comfort) are included in the definition of ‘essential’ may be optimal [11]. Alternatively, if the various factors contributing to competence are equally important, the Angoff or Borderline method may be more appropriate.
2. **How will the standard setting methods and resulting cut scores impact patients, learners and faculty?**
  Each method results in a different cut score, and the consequences of cut scores must be considered. Higher cut scores will help ensure that learners are safe and effective during patient care. However, a too‐stringent cut score will require additional resources for remediation, imposing a substantial burden on learners and faculty. Methods to investigate the impact of a cut score include (1) applying the cut score to previous assessment results (as we did in this study) to understand the impact on learners and faculty and (2) investigating the relationship between pass/fail decisions resulting from the cut score and future patient care outcomes to understand the impact on patients [3, 4, 27]. Other considerations for feasibility include the complexity and resource requirements (e.g., time, number of students) for each method as well as the understandability of each method for faculty, judges and students.

## 6 Limitations

This study only included four standard setting methods. There are also variations to the methods used in this study that could have been incorporated. For instance, during the Angoff method procedures, we did not provide judges with historical data about student performance. Although this historical assessment data should not be used a priori to influence judges' ratings, it can be used during the rating process to ensure that the resulting cut score is feasible for learners to achieve [8]. Increasing the number of judges for each method would have allowed for wider representation of multiple perspectives and more robust results. In addition, the Borderline Group and Contrasting Groups judges were the same faculty who scored each student in the actual assessment to make the standard setting process more efficient [12]. This dual role introduces bias in the results, as the checklist ratings and global ratings were performed concurrently by the same rater. Further investigation of best practices for selecting standard setting methods in clinical skills assessment is warranted.

## 7 Conclusion

In performance‐based assessments, the interpretation of testing results should include a standard setting process that promotes fair and accurate assessment decisions that ensure competent skill performance at a level appropriate to the learners' level of training. In this study, we compared four standard setting methods and applied them to a clinical skills assessment during early training in a DPT programme. This study provides specific and accessible guidance for the selection and implementation of standard setting methods to promote fair, defensible and meaningful clinical skills assessment.

## Author Contributions

Rachel S. Tappan, Heidi R. Roth and Yoon Soo Park contributed to the conceptualisation and research design. Megan L. Freeland, Elizabeth E. Holland, Heidi R. Roth and Rachel S. Tappan conducted data collection and analysis. Rachel S. Tappan prepared the original draft. All authors reviewed and edited the manuscript and approved the final manuscript.

## Ethics Statement

This study was approved by the Northwestern University Institutional Review Board (STU00220525), and all participants provided informed consent.

## Conflicts of Interest

The authors declare no conflicts of interest.

## Supporting information


**Appendix S1a:** Full list of checklist items with summary results of the judges' ratings through the Angoff and Patient Safety approaches to standard setting. The Angoff approach column indicates the mean rating with standard deviation (SD) when the judges answered the following question for each item: ‘What is the likelihood (0–100%) that a minimally competent (i.e., borderline) learner for ce1 would perform each item on an actual patient with minimum supervision, safely and effectively?’ The Patient Safety approach column indicates the number of judges who indicated that each item was essential for safety in that ‘patient safety would be at risk if the student performed the item inconsistently, ineffectively, or not at all’. Items that achieved 100% agreement and are therefore essential items are highlighted in yellow.
**Appendix S1b:** List of checklist items determined to be essential items.
**Appendix S2:** The procedures for each standard setting method are outlined with a detailed description of their application to the bed mobility clinical skills assessment in this study.

## Data Availability

The data that support the findings of this study are available from the corresponding author upon reasonable request.

## References

[1] G. J. Cizek , M. B. Bunch , and H. Koons , “Setting Performance Standards: Contemporary Methods,” Educational Measurement: Issues and Practice 23, no. 4 (2004): 31–50.

[2] American Educational Research Association , American Psychological Association , and National Council on Measurement in Education , Standards for Educational and Psychological Testing (American Educational Research Association, 2014).

[3] S. M. Downing , “Validity: On Meaningful Interpretation of Assessment Data,” Medical Education 37, no. 9 (2003): 830–837, 10.1046/j.1365-2923.2003.01594.x. 14506816

[4] D. A. Cook and M. Lineberry , “Consequences Validity Evidence: Evaluating the Impact of Educational Assessments,” Academic Medicine 91, no. 6 (2016): 785–795, 10.1097/ACM.0000000000001114. 26839945

[5] D. A. Cook , B. Zendejas , S. J. Hamstra , R. Hatala , and R. Brydges , “What Counts as Validity Evidence? Examples and Prevalence in a Systematic Review of Simulation‐Based Assessment,” Advances in Health Sciences Education: Theory and Practice 19, no. 2 (2014): 233–250, 10.1007/s10459-013-9458-4. 23636643

[6] J. H. Barsuk , E. R. Cohen , D. B. Wayne , W. C. McGaghie , and R. Yudkowsky , “A Comparison of Approaches for Mastery Learning Standard Setting,” Academic Medicine 93, no. 7 (2018): 1079–1084, 10.1097/ACM.0000000000002182. 29465449

[7] M. Homer , “Setting Defensible Minimum‐Stations‐Passed Standards in OSCE‐Type Assessments,” Medical Teacher 45, no. 10 (2023): 1163–1169, 10.1080/0142159X.2023.2197138. 37029957

[8] R. Yudkowsky , S. M. Downing , and A. Tekian , “Chapter 6: Standard Setting,” in Assessment in Health Professions Education, 2nd ed., eds. R. Yudkowsky , Y. S. Park , and S. M. Downing (Routledge Taylor & Francis, 2020): 86–105.

[9] S. M. Downing , A. Tekian , and R. Yudkowsky , “Procedures for Establishing Defensible Absolute Passing Scores on Performance Examinations in Health Professions Education,” Teaching and Learning in Medicine 18, no. 1 (2006): 50–57, 10.1207/s15328015tlm1801_11. 16354141

[10] D. B. Wayne , E. R. Cohen , and J. H. Barsuk , “Chapter 6: Standard Setting for Mastery Learning,” in Comprehensive Healthcare Simulation: Mastery Learning in Health Professions Education (Springer, 2020).

[11] R. Yudkowsky , S. Tumuluru , P. Casey , N. Herlich , and C. Ledonne , “A Patient Safety Approach to Setting Pass/Fail Standards for Basic Procedural Skills Checklists,” Simulation in Healthcare: The Journal of the Society for Simulation in Healthcare 9, no. 5 (2014): 277–282, 10.1097/SIH.0000000000000044. 25188484

[12] D. W. McKinley and J. J. Norcini , “How to Set Standards on Performance‐Based Examinations: AMEE Guide No. 85,” Medical Teacher 36, no. 2 (2014): 97–110, 10.3109/0142159X.2013.853119. 24256050

[13] R. Hays , “Standard Setting,” Clinical Teacher 12, no. 4 (2015): 226–230, 10.1111/tct.12395. 26039705

[14] S. Mortaz Hejri and M. Jalili , “Borderline Regression Method Versus Borderline Group Method,” Clinical Teacher 13, no. 1 (2016): 81, 10.1111/tct.12453. 26817752

[15] Y. S. Park , C. Kamin , D. Son , G. Kim , and R. Yudkowsky , “Differences in Expectations of Passing Standards in Communication Skills for Pre‐Clinical and Clinical Medical Students,” Patient Education and Counseling 102, no. 2 (2019): 301–308, 10.1016/j.pec.2018.09.009. 30245099

[16] M. Kane , “Choosing Between Examinee‐Centered and Test‐Centered Standard‐Setting Methods,” Educational Assessment 5, no. 3 (1998): 129–145, 10.1207/s15326977ea0503_1.

[17] A. M. Dupre , “Objectives to Assess Student Readiness for First, Full‐Time Clinical Education Experiences in Physical Therapist Education,” Journal, Physical Therapy Education 34 (2020): 242–251.

[18] H. R. Roth , E. E. Holland , L. Goh , E. Wong , W. C. McGaghie , and R. S. Tappan , “Systematic Development and Validity Evidence for a Checklist to Assess Bed Mobility Skills Among Physical Therapy Students,” Journal of Allied Health 53, no. 2 (2024): 122–129. 38834338

[19] R. Yudkowsky , Y. S. Park , M. Lineberry , A. Knox , and E. M. Ritter , “Setting Mastery Learning Standards,” Academic Medicine 90, no. 11 (2015): 1495–1500, 10.1097/ACM.0000000000000887. 26375263

[20] Commission on Accreditation in Physical Therapy Education (CAPTE) , “Standards and Required Elements for Accreditation of Physical Therapist Education Programs,” CAPTE Accreditation Handbook, (2020), accessed August 26, 2022 https://www.capteonline.org/globalassets/capte‐docs/capte‐pt‐standards‐required‐elements.pdf.

[21] “Accreditation Process Overview Fact Sheet,” The Joint Commission, (2025), retrieved January 15, 2025, https://www.jointcommission.org/resources/news‐and‐multimedia/fact‐sheets/facts‐about‐accreditation‐process‐overview/#:~:text=The%20accreditation%20process%20seeks%20to,new%20information%20and%20best%20practices.

[22] G. W. Kirwan , C. R. Clark , and M. Dalton , “Rating of Physiotherapy Student Clinical Performance: Is It Possible to Gain Assessor Consistency?,” BMC Medical Education 19, no. 1 (2019): 32, 10.1186/s12909-019-1459-4. 30678662 PMC6346544

[23] 2023 Accreditation Council for Occupational Therapy Education (ACOTE®) , “2023 Accreditation Council for Occupational Therapy Education (ACOTE®) Standards and Interpretive Guide,” Accreditation Council for Occupational Therapy Education, (2023), retrieved January 17, 2025, https://acoteonline.org/accreditation‐explained/standards/.

[24] “Standards and Criteria for Academic Quality of Post‐Secondary and Higher Degree Programs in Nursing,” Accreditation Commission on Nursing Education (ECEN), (2023), retrieved January 17, 2025, https://resources.acenursing.org/space/SAC/1825603752/STANDARD+4+‐+Curriculum.

[25] K. Stevens , H. Henderson , K. Hawthorne , and J. Carlson , “A Comparison of Methods for Setting Passing Scores in Standardized Simulated Patient Experiences in Physical Therapist Education,” Journal, Physical Therapy Education 27, no. 3 (2013): 78–81.

[26] American Educational Research Association , American Psychological Association , and National Council on Measurement in Education , “Scores, Scales, Norms, Score Linking, and Cut Scores,” in *Standards for Educational and Psychological Testing* (American Educational Research Association, 2014), 95–109.

[27] D. A. Cook and R. Hatala , “Validation of Educational Assessments: A Primer for Simulation and Beyond,” Advances in Simulation 1 (2016): 31, 10.1186/s41077-016-0033-y. 29450000 PMC5806296

