# Supplementary material for: Comparison of Standard Setting Procedures to Establish Defensible Passing Standards for Clinical Skills Assessment: Angoff, Borderline Group, Contrasting Groups and Patient Safety Methods
Source: Clin Teach. 2025 Sep 3;22(5):e70198. doi: 10.1111/tct.70198 (PMC12409087; doi:10.1111/tct.70198)
Supplement: Supplementary file 1 — Appendix S1a: Full list of checklist items with summary results of the judges' ratings through the Angoff and Patient Safety approaches to standard setting. The Angoff approach column indicates the mean rating with standard deviation (SD) when the judges answered the following question for each item: ‘What is the likelihood (0–100%) that a minimally competent (i.e., borderline) learner for ce1 would perform each item on an actual patient with minimum supervision, safely and effectively?’ The Patient Safety approach column indicates the number of judges who indicated that each item was essential for safety in that ‘patient safety would be at risk if the student performed the item inconsistently, ineffectively, or not at all’. Items that achieved 100% agreement and are therefore essential items are highlighted in yellow. Appendix S1b: List of checklist items determined to be essential items. Appendix S2: The procedures for each standard setting method are outlined with a detailed description of their application to the bed mobility clinical skills assessment in this study. [file TCT-22-e70198-s001.docx]

Appendix 1a. Full list of checklist items with summary results of the judges’ ratings through the Angoff and Patient Safety approaches to standard setting. The Angoff approach column indicates the mean rating with standard deviation (SD) when the judges answered the following question for each item: “What is the likelihood (0-100%) that a minimally competent (i.e., borderline) learner for CE1 would perform each item on an actual patient with minimum supervision, safely and effectively?” The Patient Safety approach column indicates the number of judges who indicated that each item was essential for safety in that “patient safety would be at risk if the student performed the item inconsistently, ineffectively, or not at all.” Items that achieved 100% agreement and are therefore essential items are highlighted in yellow.

| **Bed Mobility Checklist Items** | **Angoff method:**  **Mean % (SD)** | **Patient Safety method: Frequency (%)** |
| --- | --- | --- |
| 1. SPT introduces self by full name, student physical therapist. | 95.0% (5.8) | 0 (0.0%) |
| 2. SPT confirms patient identification by confirming patient’s full name and date of birth. | 87.5% (15.0) | 8 (88.9%) |
| 3. SPT confirms with patient how they would like to be addressed (name and/or pronouns). | 71.3% (14.4) | 0 (0.0%) |
| 4. SPT provides overview and rationale of tasks. | 57.5% (9.6) | 0 (0.0%) |
| 5. SPT confirms patient agreement with plan. | 57.5% (9.6) | 0 (0.0%) |
| 6. Hand Hygiene: SPT sanitizes hands before touching patient. | 80.0% (14.1) | 8 (88.9%) |
| 7. Precautions: SPT reviews any relevant precautions with the patient (e.g., THR, spine, etc.) and confirms patient understanding. | 70.0% (21.6) | 9 (100.0%) |
| 8. Instructions: SPT provides clear and effective instructions prior to initiating movement (e.g., verbal, written, and/or demonstration). | 62.5% (9.6) | 2 (22.2%) |
| Skill 1: Rolling |  |  |
| 9. Set-up: SPT sets up environment to optimize patient performance. | 67.5% (17.1) | 1 (11.1%) |
| 10. SPT Position: SPT positions self (therapist) to facilitate/assist patient movement and maximize patient safety during rolling. | 66.3% (7.5) | 4 (44.4%) |
| 11. SPT Assistance: SPT allows patient to do as much of the movement as possible and assists patient with roll only as needed. | 60.0% (11.5) | 0 (0.0%) |
| 12. Body Part Support: SPT protects/supports patient’s joints and skin sufficiently (e.g., avoids skin shearing and friction) during movement of body parts. | 71.3% (2.5) | 4 (44.4%) |
| 13. Lines and Tubes: SPT manages lines and tubes during movement with no undue pressure or tension placed on any lines or tubes and ensures safe placement of equipment (e.g., Foley below level of bladder). | 71.3% (21.7) | 9 (100.0%) |
| 14. Precautions: SPT maintains all relevant movement precautions. | 70.0% (8.2) | 9 (100.0%) |
| 15. Fall Risk: SPT minimizes patient fall risk throughout skill. | 81.3% (13.1) | 9 (100.0%) |
| Skill 2: Lying Down to Sitting Up |  |  |
| 16. Method: SPT selects appropriate method of transition from lying down to sitting (e.g., via rolling, via long sit) that optimizes patient participation and independence. | 67.5% (12.6) | 0 (0.0%) |
| 17. Set-up: SPT sets up environment to optimize patient performance. | 61.3% (13.1) | 1 (11.1%) |
| 18. SPT Position: SPT positions self (therapist) to facilitate/assist patient movement and maximize patient safety. | 63.8% (7.5) | 4 (44.4%) |
| 19. SPT Assistance: SPT allows patient to do as much of the movement as possible and assists patient with sitting up only as needed. | 57.5% (9.6) | 0 (0.0%) |
| 20. Body Part Support: SPT protects/supports patient’s joints and skin sufficiently (e.g., avoids skin shearing and friction) during movement of body parts. | 71.3% (2.5) | 4 (44.4%) |
| 21. Lines and Tubes: SPT manages lines and tubes during movement with no undue pressure or tension placed on any lines or tubes and ensures safe placement of equipment (e.g., Foley below level of bladder). | 63.8% (16.0) | 9 (100.0%) |
| 22. Precautions: SPT maintains all relevant movement precautions. | 70.0% (8.2) | 9 (100.0%) |
| 23. Fall Risk: SPT minimizes patient fall risk throughout skill. | 70.0% (14.1) | 9 (100.0%) |
| Skill 3: Sitting Up to Lying Down |  |  |
| 24. Method: SPT selects appropriate method of transition from sitting up to lying down (e.g., via side lying or down on elbow) that optimizes patient participation and independence. | 67.5% (12.6) | 0 (0.0%) |
| 25. Set-up: SPT sets up environment to optimize patient performance. | 61.3% (13.1) | 1 (11.1%) |
| 26. SPT Position: SPT positions self (therapist) to facilitate/assist patient movement and maximize patient safety. | 63.8% (7.5) | 4 (44.4%) |
| 27. SPT Assistance: SPT allows patient to do as much of the movement as possible and assists patient with lying down only as needed. | 63.8% (11.1) | 0 (0.0%) |
| 28. Body Part Support: SPT protects/supports patient’s joints and skin sufficiently (e.g., avoids skin shearing and friction) during movement of body parts. | 61.3% (13.1) | 4 (44.4%) |
| 29. Lines and Tubes: SPT manages all lines and tubes during transition from sitting up to lying down without undue pressure or tension placed on any lines or tubes and ensures safe placement of equipment (e.g., Foley below level of bladder). | 61.3% (10.3) | 9 (100.0%) |
| 30. Precautions: SPT maintains all relevant movement precautions. | 70.0% (8.2) | 9 (100.0%) |
| 31. Fall Risk: SPT minimizes patient fall risk throughout skill. | 75.0% (12.9) | 9 (100.0%) |
| Skill 4: Final Position |  |  |
| 32. Body Part Support: Final patient position supports and protects any vulnerable body parts, for instance, related to skin breakdown, edema, or medical condition. | 75.0% (4.1) | 5 (55.6%) |
| 33. Lines and Tubes: Final patient position provides appropriate placement of lines and tubes with no undue pressure or tension placed on any lines or tubes and ensures safe placement of equipment (e.g., Foley below level of bladder). | 77.5% (8.7) | 9 (100.0%) |
| 34. Precautions: Final patient position maintains all relevant movement precautions. | 78.8% (14.4) | 9 (100.0%) |
| 35. Comfort: Final patient position maximizes patient comfort, confirmed with patient (e.g., verbal, nonverbal, written feedback). | 72.5% (15.0) | 2 (22.2%) |
| 36. Fall Risk: Final patient position minimizes patient fall risk. | 77.5% (16.6) | 9 (100.0%) |
| Overall Encounter |  |  |
| 37. Draping: SPT provides appropriate draping for modesty throughout the task and in final patient position. | 55.0% (5.8) | 0 (0.0%) |
| 38. Body Mechanics: SPT uses appropriate body mechanics and equipment set-up to minimize risk of therapist injury. | 57.5% (5.8) | 1 (11.1%) |
| 39. Communication: SPT’s verbal (e.g., volume and tone) and nonverbal communication is appropriate for patient scenario throughout the encounter (e.g., considers cognition and communication abilities and psychosocial situation). | 55.0% (5.8) | 0 (0.0%) |
| 40. Feedback: SPT provides individual feedback to patient on their performance. | 52.5% (9.6) | 0 (0.0%) |
| 41. Session Management: SPT leads session without long pauses or >2 time-outs. | 55.0% (5.8) | 0 (0.0%) |
| 42. Closure: SPT provides closure to end session. | 73.8% (4.8) | 0 (0.0%) |
| 43. Hand Hygiene: SPT sanitizes hands after end of session, before exiting the room. | 73.8% (9.5) | 4 (44.4%) |
| 44. PPE: SPT uses appropriate PPE (e.g., mask, eye protection, gloves) throughout the patient encounter. | 78.8% (16.5) | 9 (100.0%) |
| Documentation |  |  |
| 45. SPT documents bed mobility (movements). | 71.3% (8.5) | 1 (11.1%) |
| 46. SPT documents level of assist, including cues. | 68.8% (13.1) | 1 (11.1%) |
| 47. SPT documents final patient position. | 56.3% (12.5) | 0 (0.0%) |
| 48. SPT documents patient response. | 58.8% (10.3) | 1 (11.1%) |

*Checklist items were originally published in:

Roth HR, Holland EEH, Goh L, Wong E, McGaghie WC, Tappan RS. Systematic development and validity evidence for a checklist to assess bed mobility skills among physical therapy students. *J Allied Health.* 2024;53(2):122-129.

Appendix 1b. List of checklist items determined to be essential items.

| Bed Mobility Checklist – Essential Items |
| --- |
| **7. Precautions: SPT reviews any relevant precautions with the patient (e.g., THR, spine, etc.) and confirms patient understanding.** |
| Skill 1: Rolling |
| **13. Lines and Tubes: SPT manages lines and tubes during movement with no undue pressure or tension placed on any lines or tubes and ensures safe placement of equipment (e.g., Foley below level of bladder).** |
| **14. Precautions: SPT maintains all relevant movement precautions.** |
| **15. Fall Risk: SPT minimizes patient fall risk throughout skill.** |
| Skill 2: Lying Down to Sitting Up |
| **21. Lines and Tubes: SPT manages lines and tubes during movement with no undue pressure or tension placed on any lines or tubes and ensures safe placement of equipment (e.g., Foley below level of bladder).** |
| **22. Precautions: SPT maintains all relevant movement precautions.** |
| **23. Fall Risk: SPT minimizes patient fall risk throughout skill.** |
| Skill 3: Sitting Up to Lying Down |
| **29. Lines and Tubes: SPT manages all lines and tubes during transition from sitting up to lying down without undue pressure or tension placed on any lines or tubes and ensures safe placement of equipment (e.g., Foley below level of bladder).** |
| **30. Precautions: SPT maintains all relevant movement precautions.** |
| **31. Fall Risk: SPT minimizes patient fall risk throughout skill.** |
| Skill 4: Final Position |
| **33. Lines and Tubes: Final patient position provides appropriate placement of lines and tubes with no undue pressure or tension placed on any lines or tubes and ensures safe placement of equipment (e.g., Foley below level of bladder).** |
| **34. Precautions: Final patient position maintains all relevant movement precautions.** |
| **36. Fall Risk: Final patient position minimizes patient fall risk.** |
| Overall Encounter |
| **44. PPE: SPT uses appropriate PPE (e.g., mask, eye protection, gloves) throughout the patient encounter.** |

Appendix 2:

The procedures for each standard setting method are outlined below with a detailed description of their application to the bed mobility clinical skills assessment in this study.

***Angoff Method***^1,2^

*Step 1: Select a Standard Setting Method*

The Angoff method is relatively easy to understand and implement yet still provides an evidence-based cut score. In our study, we selected the Angoff method because of its widespread use in health professions education and ease of administration.^2^

*Step 2: Describe Performance Categories*

Expectations for the learners’ level of performance should be clearly defined based on the inferences that will be made about the learners’ abilities as a result of their performance on the assessment. How competent does the learner need to be and for what context?

In our study, the student physical therapists (SPTs) were preparing for entrance into their first full-time clinical experience (CE1), which requires them to be able to safely and effectively supervise and assist individuals with mobility skills (e.g., moving in bed, transferring from one surface to another, locomotion via gait or wheelchair propulsion), and perform basic physical therapy tests and measures for noncomplex patients.^3^ The expectations for this assessment, therefore, were that the cut score would be consistent with a SPT who is performing bed mobility skills at a minimally competent level for entrance into CE1.

*Step 3: Select Judges*

For criterion-based methods such as those used in this study, the standard is based on the judges’ clinical expertise in the knowledge and skills being assessed, familiarity with typical performance at the given level of student training, and ability to follow standard setting procedures accurately and fairly. Therefore, the selection of credible judges is paramount. For the Angoff method, judge panels typically consist of 5-12 members.^1^

In our study, the judges were physical therapists with experience teaching first-year SPTs from the Northwestern University DPT program in the classroom or the clinic. Judge selection aimed for diverse representation of teaching roles including educators employed by the university whose primary role is teaching (core faculty) as well as clinicians employed by outside clinics who assist with classroom teaching (associated faculty) and who supervise SPTs during full-time clinical experiences (clinical instructors).

*Steps 4-5: Standard Setting Meeting*

Steps 4-5 occur during a meeting where judges undergo training (Step 4) and then provide ratings (Step 5) that are subsequently used for calculation of the cut score.

In our study, we conducted a single, online two-hour meeting using Zoom video conference software (Zoom Video Communications, Inc., San Jose, CA) to complete the Angoff and Patient Safety method procedures with the first hour devoted to the Angoff Method. During the meeting, judges’ ratings were entered and compiled in real time via Google Sheets software (Google, Mountain View, CA).

*Step 4: Train Judges*

In this step, judges undergo training that includes standard setting purpose and key concepts, procedures for the specific method(s) used, and key definitions such as the performance categories from Step 2.^1^

In our study, the judges were familiarized with the purpose and procedures of the bed mobility skills assessment,^4^ the performance expectations for this assessment (described in Step 2), the Commission on Accreditation in Physical Therapy Education’s (CAPTE’s) accreditation standards for clinical skills assessment,^5^ and the Angoff method procedures described in Step 5. This training occurred through written materials sent to judges prior to the standard setting meeting and a brief (5-10 minute) discussion at the beginning of the standard setting meeting.

*Step 5: Standard Setting*

In the Angoff method, each item in the assessment checklist is evaluated individually with the following steps^1,2^:

1. Judges develop a shared mental model for borderline performance by discussing example characteristics of what they would deem to be borderline performance on the assessment based on their experiences and expertise.
2. Each judge estimates what percentage of learners who are borderline for proceeding to the next stage of training would perform each item correctly.
3. The judges discuss their responses for individual checklist items including providing rationale for ratings when there is wide variation. They may also review previous examinee performance data if it is available to promote familiarity with typical performance. The goal of the discussion is to inform the judges’ ratings, so that they are considering varied perspectives in their decision-making.
4. Judges are then allowed to adjust their estimates if desired.
5. The mean of all judges’ estimates for all items is then the cut score for the assessment.

In our study, judges answered the following question to complete their estimates for each checklist item, “What is the likelihood that a minimally competent (i.e., borderline) learner entering CE1 would perform each item correctly?” The mean, standard deviation (SD), and range of ratings for each item were shared with the group, and judges discussed these ratings. Judges were then given the opportunity to change any of their answers. The mean of the judges’ final ratings was then the cut score for the total score on the assessment, defined as the percentage of all items that receive a “Yes” rating.

***Patient Safety Method***^1,6^

*Step 1: Select a Standard Setting Method*

The Patient Safety method was developed for use with clinical skills assessments, where some items are more important than others and therefore held to a higher standard.^6^

In our study, the decisions about SPT readiness for patient care that resulted from the assessment aligned well with the Patient Safety method’s incorporation of clinical and accreditation priorities of patient safety.^5^

*Steps 2 and 3: Describe Performance Categories and Select Judges*

Steps 2 and 3 are the same for the Angoff method and the Patient Safety method.

*Steps 4-5: Standard Setting Meeting*

Steps 4-5 occur during a meeting where judges undergo training (Step 4) and then provide ratings (Step 5) that are subsequently used for calculation of the cut score. In this study, the Patient Safety method procedures were performed during the second hour of the previously described two-hour online meeting.

*Step 4: Train Judges*

As with the Angoff method, judges undergo training that includes the standard setting purpose and key concepts, procedures for the specific method(s) used, and key definitions such as the performance categories from Step 2.^1^ This training occurred through written materials sent to judges prior to the standard setting meeting and a brief (5-10 minute) discussion at the beginning of the standard setting meeting.

In this study, the judges were familiarized with the purpose and procedures of the bed mobility skills assessment,^4^ the performance expectations and categories for this assessment (described in Step 2), CAPTE’s accreditation standards for clinical skills assessment,^5^ and the Patient Safety method procedures described in Step 5.

*Step 5: Standard Setting*^1,6^

The Patient Safety method includes a separate standard for essential assessment items, such as those related to patient and/or clinician safety, patient outcome, and/or patient comfort, and non-essential assessment items that must be achieved for a student to pass the assessment.

Identifying essential items: Items are categorized as essential and non-essential with the following steps:

1. The criteria (e.g., patient and/or clinician safety, patient outcome, and/or patient comfort) for essential items are identified based on the intended use and interpretation of the assessment. The level of agreement required to deem an assessment item as “essential” is also determined. These decisions are typically made prior to the standard setting meeting.
2. In the standard setting meeting, each judge rates which assessment items are essential and non-essential.
3. The judges discuss their ratings, including providing rationale for ratings. The goal of the discussion is to inform the judges’ ratings, so that they are considering varied perspectives in their decision-making.
4. Judges are then allowed to adjust their ratings if desired.
5. Items that meet the pre-determined level of agreement required to deem an item as “essential” become the essential items. The remaining items are non-essential.

Generating cut scores: The judges generate separate cut scores for the essential items and the non-essential items. This step may be completed at the item level or at the category level.

1. Item-Level Cut Scores:
   - 1. For each item, each judge answers the question “What is the likelihood that a minimal competent learner would perform this item correctly?”
     2. Judges discuss their ratings, including providing rationale for ratings. The goal of the discussion is to inform the judges’ ratings, so that they are considering varied perspectives in their decision-making.
     3. Judges are allowed to adjust their ratings if desired.
     4. Judges’ ratings are averaged for the essential items and non-essential items to create a single conjunctive minimum passing standard where the learner must achieve the cut-off for the essential items and the cut-off for the non-essential items.
2. Category-Level Cut Scores:
   - 1. For each category, each judge answers the questions “What percentage of essential items would a minimally competent learner perform correctly? What percentage of non-essential items would a minimally competent learner perform correctly?”
     2. Judges discuss their ratings, including providing rationale for ratings. The goal of the discussion is to inform the judges’ ratings, so that they are considering varied perspectives in their decision-making.
     3. Judges are allowed to adjust their ratings if desired.
     4. Judges’ ratings are averaged for each category to create a single conjunctive minimum passing standard where the learner must achieve the cut-off for the essential items and the cut-off for the non-essential items.

In our study, we determined *a priori* that the essential items would be those that were essential specifically for patient safety. The judges rated which checklist items were essential for patient safety according to the following criterion: “Critical items where patient safety would be at risk if the student performed the item inconsistently, ineffectively, or not at all.” with 100% agreement amongst judges required for an item to be named an “essential item.” Then, the frequencies of judges’ ratings for each item were shared with the group. Judges discussed their ratings and had the opportunity to modify their responses. Items that reached 100% agreement as critical for safety were determined to be essential items. All other items were non-essential.

Next, judges determined separate passing scores for essential and non-essential items by answering the following questions:

1. What percentage of essential/critical safety items would a minimally competent learner entering CE1 perform correctly?
2. What percentage of non-essential items (i.e., items not critical for patient safety) would a minimally competent learner entering CE1 perform correctly?

The mean, SD, and range of ratings for each question were shared with and discussed by the group, and judges were given the opportunity to change any of their answers.

Two conjunctive cut scores (i.e., with separate cut-off scores for the essential items and non-essential items) were then calculated via two methods: 1) category-based method: calculating the mean of the judges’ answers to the two questions listed above, and 2) item-based method: calculating the mean of the judges’ item-by-item estimates that had already been gathered during the Angoff standard setting process.

***Borderline Group Method***^1,2^

*Step 1: Select a Standard Setting Method*

The Borderline method uses ratings of the learners’ global performance on the assessment to determine the cut score. This method doesn’t require a separate standard setting meeting, though it does require a substantial sample of learners with borderline performance (i.e., not clearly adequate but also not clearly inadequate) to be able to calculate a cut score.^1,2^

In our study, we selected the Borderline Group method because of its incorporation of actual SPT performance in the standard setting process and the relatively large sample size of examinees

*Step 2: Describe Performance Categories*

Step 2 is the same as for the Angoff and Patient Safety methods.

*Step 3: Select Judges*

In the Borderline Group method, an expert rater provides a global rating of each learner’s performance. In our study the judges were the faculty raters who also rated SPT performance during the assessment.

*Step 4: Train Judges*

In this step, the judges undergo training that includes standard setting purpose and key concepts, procedures for the specific method(s) used, key definitions such as the performance categories from Step 2.^1^

In our study, the judges were already familiar with the purpose and procedures of the bed mobility skills assessment^4^ due to their faculty role in the course. In addition, the judges watched a seven-minute online training video before the assessment and had an opportunity to ask questions in-person immediately prior to the assessment. The online training video included a rationale for undergoing standard setting procedures, performance expectations for this assessment (e.g., cut score consistent with a student who is “minimally competent” for the first full-time clinical experience), CAPTE accreditation standards for clinical skills assessment,^5^ and the standard setting procedures described in Step 5, including the definition of “borderline” performance.

*Step 5: Standard Setting*

In the Borderline Group method, the cut score is based on the assessment scores of learners who are rated as having a “borderline” performance by faculty raters.^1,2^ The steps are as follows:

1. The faculty raters make a judgement about each learner’s overall performance immediately after the learner has completed the assessment by answering the following two questions:

1. Would you rate this learner’s performance as “pass” or “fail”?

2. Was this learner’s performance on the borderline between Pass and Fail, i.e., “marginal pass” or “marginal fail”?

1. The cut score is calculated as the mean or median checklist score of the learners who were rated as having borderline performance.

In our study, each SPT completed the bed mobility skills assessment with faculty scoring their performance on the assessment checklist. At the end of the assessment, the faculty raters immediately gave a global rating of each SPT’s performance by answering “Yes” or “No” to the question above. The cut score was then determined by calculating the mean checklist score for the SPT for whom the answer was “Yes.”

***Contrasting Groups Method***^1,2^

*Step 1: Select a Standard Setting Method*

The Contrasting Groups method uses ratings of the learners’ global performance on the assessment to determine the cut score. This method doesn’t require a separate standard setting meeting.^1,2^

In our study, we selected the Contrasting Groups method because of its incorporation of actual SPT performance in the standard setting process.

*Step 2: Describe Performance Categories*

Step 2 is the same as for the Angoff and Patient Safety methods.

*Step 3: Select Judges*

In the Contrasting Groups method, an expert rater provides a global rating of each learner’s performance. In our study the judges were the faculty raters who also rated SPT performance during the assessment.

*Step 4: Train Judges*

In this step, the judges undergo training that includes standard setting purpose and key concepts, procedures for the specific method(s) used, key definitions such as the performance categories from Step 2.^1^

In our study, the judges were already familiar with the purpose and procedures of the bed mobility skills assessment^4^ due to their faculty role in the course. In addition, the judges watched the seven-minute online training video described in the Borderline Group method section before the assessment and had an opportunity to ask questions in-person immediately prior to the assessment.

*Step 5: Standard Setting*

In the Contrasting Groups method, the cut score is based on the assessment score that best discriminates between learners who are assigned to two groups (“pass” and “fail”) based on the judges’ global ratings of each learner’s performance. In this study, the global ratings of “pass” and “fail” were based on judges’ determination of whether the learner’s performance was consistent with at least “minimally competent” for CE1 (“pass”) or not (“fail.”)^1,2^ The steps are as follows:

1. The faculty raters make a judgement about each learner’s overall performance after the learner has completed the assessment by answering the following question: Would you rate this learner’s performance as “pass” or “fail”?
2. The checklist score distribution for the “pass” and “fail” groups are graphed.
3. The cut score is set at the intersection between the two distributions.

In our study, each SPT completed the bed mobility skills assessment with faculty scoring their performance on the assessment checklist. At the end of the assessment, the faculty raters immediately gave a global rating of each SPT’s performance by answering “Yes” or “No” to the question above. The cut score was then determined as described in Steps 5a-c above. See Figure 1 in this Appendix for the distribution graph.

Figure 1


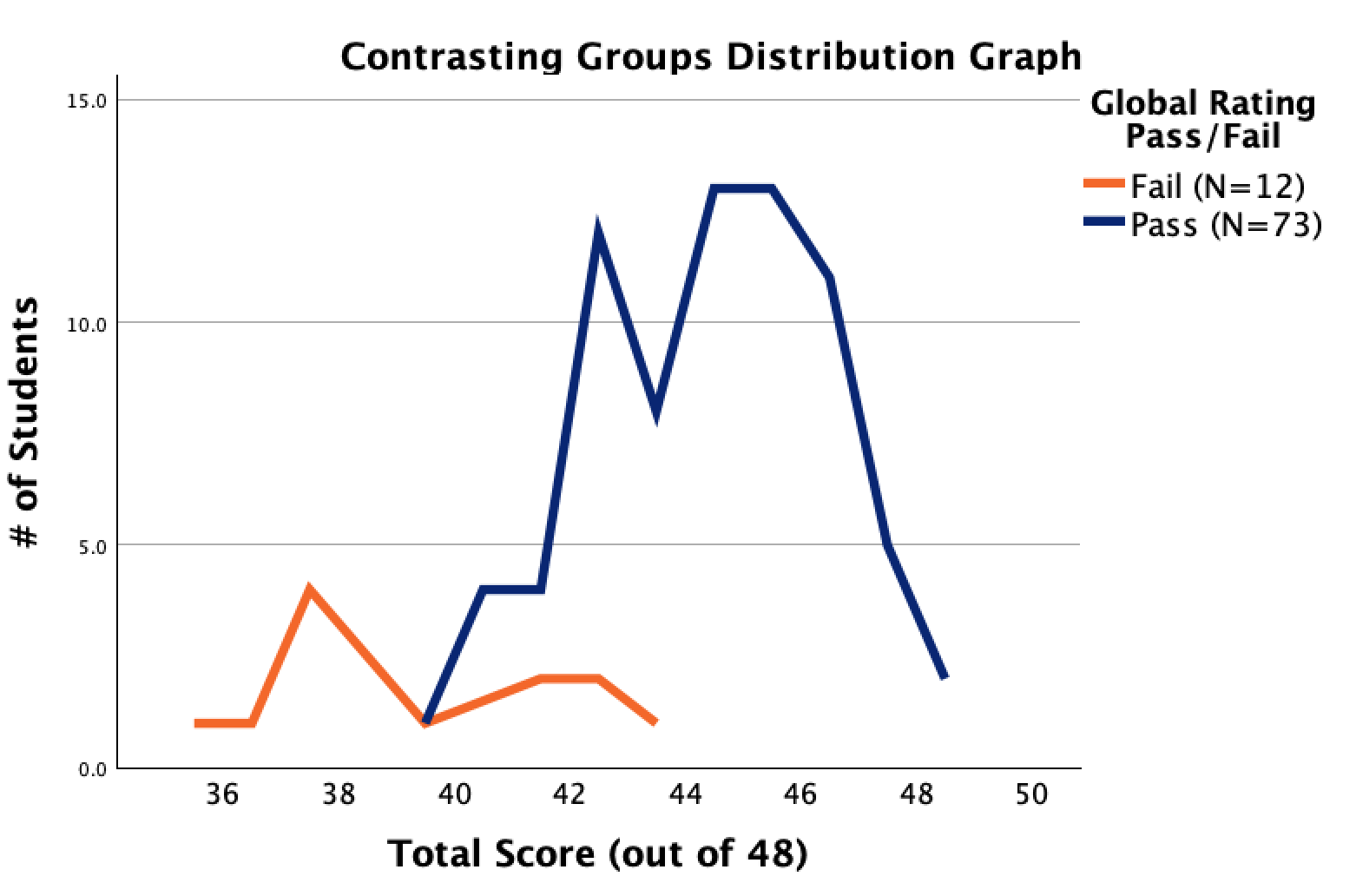


References

1. Yudkowsky R, Downing SM, Tekian A. Chapter 6: Standard Setting. In: Yudkowsky R, Park YS, Downing SM, eds. *Assessment in Health Professions Education*. 2nd ed. Routledge Taylor & Francis; 2020:86-105.

2. Downing SM, Tekian A, Yudkowsky R. Procedures for establishing defensible absolute passing scores on performance examinations in health professions education. *Teach Learn Med*. 2006;18(1):50-57. doi:10.1207/s15328015tlm1801_11

3. Dupre AM. Objectives to assess student readiness for first, full-time clinical education experiences in physical therapist education. *J Phys Ther Educ*. 2020;34:242-251.

4. Roth HR, Holland EE, Goh L, Wong E, McGaghie WC, Tappan RS. Systematic development and validity evidence for a checklist to assess bed mobility skills among physical therapy students. *J Allied Health*. 2024;53(2):122-129.

5. Commission on Accreditation in Physical Therapy Education (CAPTE). *Standards and Required Elements for Accreditation of Physical Therapist Education Programs. CAPTE Accreditation Handbook*.; 2020. Accessed August 26, 2022. https://www.capteonline.org/globalassets/capte-docs/capte-pt-standards-required-elements.pdf

6. Yudkowsky R, Tumuluru S, Casey P, Herlich N, Ledonne C. A patient safety approach to setting pass/fail standards for basic procedural skills checklists. *Simul Healthc J Soc Simul Healthc*. 2014;9(5):277-282. doi:10.1097/SIH.0000000000000044
